# Effects of variants of 50 genes on diabetes risk among the Chinese population born in the early 1960s

**DOI:** 10.1111/1753-0407.12922

**Published:** 2019-04-25

**Authors:** Chao Song, Meng Wang, Hongyun Fang, Weiyan Gong, Deqian Mao, Caicui Ding, Qiqi Fu, Ganyu Feng, Zheng Chen, Yanning Ma, Yecheng Yao, Ailing Liu

**Affiliations:** ^1^ Chinese Center for Disease Control and Prevention National Institute for Nutrition and Health Beijing China

**Keywords:** diabetes, genetic susceptibility, single nucleotide polymorphism, 糖尿病, 基因易感性, 单核苷酸多态性

## Abstract

**Background:**

Genome‐wide association studies have identified loci that significantly increase diabetes risk. This study explored the genetic susceptibility in relation to diabetes risk in adulthood among a Chinese population born in the early 1960s.

**Methods:**

In all, 2129 subjects (833 males, 1296 females) were selected from the cross‐sectional 2010 to 2012 China National Nutrition and Health Survey. Fifty diabetes‐related single nucleotide polymorphisms (SNPs) were detected. Two diabetes genetic risk scores (GRSs) based on the 50 diabetes‐predisposing variants were developed to examine the association of these SNPs with diabetes risk.

**Results:**

Associations were found between diabetes risk and SNPs in the *MTNR1B* (rs10830963), *KLHDC5* (rs10842994), *GRK5* (rs10886471), *cyclindependentkinase 5 regulatory subunit associated protein 1* (rs10946398), *adaptorrelated protein complex 3 subunit sigma 2* (rs2028299), *diacylglycerol kinase beta/transmembrane protein 195* (rs2191349), *SREBF chaperone* (rs4858889), *ankyrin1* (rs516946), *RAS guanyl releasing protein 1* (rs7403531), and *zinc finger AN1‐type containing 3* (rs9470794) genes. As a continuous variable, with a 1‐point increase in the GRS or weighted (w) GRS, fasting plasma glucose (FPG) increased 0.045 and 0.044 mM, respectively (*P <* 0.001 for both), after adjusting for confounders. Both GRS and wGRS showed an association with diabetes, with a multivariable‐adjusted odds ratio (95% confidence interval) of 1.09 (1.00‐1.19) and 1.12 (1.03‐1.22), respectively, among all subjects. No significant associations were found between the GRS or wGRS and impaired fasting glucose or impaired glucose tolerance.

**Conclusions:**

The data suggest the association of 10 SNPs and the GRS or wGRS with diabetes risk. Genetic susceptibility to diabetes may synergistically affect the risk of diabetes in adulthood.

## Graphical abstract

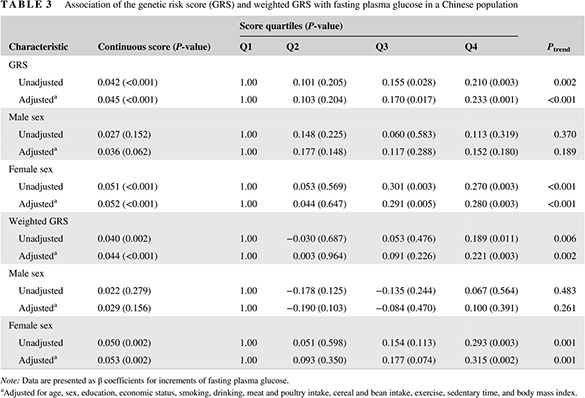

**Highlights**

- Ten single nucleotide polymorphisms were found to be associated with diabetes risk.
- There were significant associations of diabetes genetic risk scores and weighted diabetes genetic risk scores with fasting plasma glucose and diabetes.

## 摘要

**背景:**

全基因关联性分析研究发现了能显著增加糖尿病发病风险的位点。本研究旨在探讨20世纪60年代初出生的中国人群糖尿病相关基因与糖尿病发病风险的关系。

**方法:**

从2010‐2012年中国居民营养与健康状况监测的研究对象中选取2129名在20世纪60年代初出生的中国人群作为研究对象，其中男性833名，女性1296名。对研究对象进行50个糖尿病相关的单核苷酸多态性(single nucleotide polymorphism，SNP)进行检测。然后用糖尿病相关变异计算得到两种糖尿病基因评分(GRS/weighted GRS)，分析其与糖尿病发病风险的关系。

**结果:**

研究发现MTNR1B (rs10830963)、KLHDC5 (rs10842994)、GRK5 (rs10886471)、CDKAL1 (rs10946398)、AP3S2 (rs2028299)、DGKB/TMEM195 (rs2191349)、 SCAP (rs4858889)、ANK1 (rs516946)、RASGRP1 (rs7403531)、 ZFAND3 (rs9470794)与糖尿病发病风险之间存在相关性。糖尿病基因评分作为连续变量时，校正混杂因素后，糖尿病基因评分(GRS)和加权GRS(wGRS)每增加一个单位，空腹血糖浓度分别增加0.045 mmol/L和0.044 mmol/L(两者均为*P*<0.001)。GRS和wGRS均与糖尿病患病风险有关(分别为OR=1.09，95% CI: 1.00‐1.19；OR=1.12，95% CI: 1.03‐1.22)。未发现GRS及wGRS与空腹血糖异常和葡萄糖耐量受损发生相关。

**结论:**

研究发现10个SNP以及GRS或wGRS与糖尿病发病风险相关。糖尿病基因易感性会协同影响成年糖尿病的发病风险。

## 1 INTRODUCTION

Diabetes is a serious public health problem. The number of cases and the prevalence of diabetes have been steadily increasing over the past few decades. Globally, an estimated 422 million adults were living with diabetes in 2014.^1^ According to the Report on Chinese Residents' Chronic Disease and Nutrition (2015),^2^ the prevalence of diabetes among Chinese adults aged 18 years or older had increased from 4.2% in 2002 to 9.7% in 2012. Although environmental factors such as diet and lifestyle have clearly contributed to the recent rise in the prevalence of diabetes, there is increasing evidence that common variants in the human genome contribute to the development of diabetes.^3^, ^4^, ^5^, ^6^, ^7^

To date, more than 100 single nucleotide polymorphisms (SNPs) have been identified as diabetes risk loci in different ethnic populations by genome‐wide association studies (GWAS).^8^ However, few studies have investigated many loci in a representative Chinese population. So, in this study we used data from the China National Nutrition and Health Survey (CNNHS) 2010 to 2012 to evaluate genetic susceptibility by combining the 61 SNPs identified from recent GWAS.^6^, ^9^, ^10^, ^11^, ^12^

## 2 METHODS

### 2.1 Research design and subjects

The CNNHS 2010 to 2012 was a national representative cross‐sectional study conducted by the National Institute for Nutrition and Health (NINH), Chinese Center for Disease Control and Prevention (China CDC). The 2010 to 2012 survey covered all 31 provinces, autonomous regions, and municipalities throughout China (except Taiwan, Hong Kong, and Macao). According to data provided by the China National Bureau of Statistics, the country was classified into four strata based on economy and social development: large cities, medium and small cities, ordinary rural areas, and poor rural areas.^13^ Subjects were recruited to the study using a stratified multistage cluster and probability proportional to size sampling design, which has been described previously.^14^ Questionnaires were used to collect information on demographic characteristics. Blood samples were collected from subjects.

For the present study, subjects born in 1960, 1961, and 1963 were selected. The exclusion criteria were unqualified blood sample, failure of DNA extraction, abnormal gene detection results, incomplete basic information, and the presence of liver, kidney, and heart diseases and cancer. In addition, subjects who had been diagnosed with diabetes and changed their lifestyle before the study recruitment were excluded from the study. This left 2219 subjects who were included in the present study.

The protocols of the 2010 to 2012 CNNHS and Fetal Origin Hypothesis of Diabetes: Thrifty Genotype Hypothesis or Thrifty Phenotype studies were approved by the Ethics Committee of the NINH, China CDC (2013‐018, 2013‐010). Signed consent was obtained from all subjects.

### 2.2 Genotyping

Originally, 61 SNPs that had a nominal to strong association with diabetes in recently published GWAS were selected.^6^, ^10^, ^11^, ^12^, ^15^, ^16^, ^17^, ^18^, ^19^ A mass array system (Agena, San Diego, California) was used to detect the genotypes of 61 diabetes‐related SNPs. No significant departures from Hardy‐Weinberg equilibrium (HWE) were detected among subjects without diabetes (Table S1), which suggested that the subjects was representative of the population generally. At the individual level, blood samples whose call rates were < 50% were removed from analysis. At the SNP level, SNPs were excluded if their call rate was <80% and/or their *P*‐value for HWE was <0.0001 in subjects without diabetes. Thus, 2129 subjects and 50 SNPs were finally included in the analysis.

### 2.3 Assessment of variables

Information about demographic characteristics, dietary factors, smoking and drinking status, family history of diabetes, exercise data, and anthropometric data was derived from the questionnaires. Self‐reported education levels were divided into three categories: (a) illiteracy to primary school; (b) junior middle school; and (c) senior high school or higher. Current economic status was assessed on the per capita annual income of households in 2011, and was divided into three levels: <20 000, 20 000‐40 000 RMB, >40 000 Yuan. Smoking and drinking status was classified as “yes” or “no”.

A validated semiquantitative food frequency questionnaire and 24‐hour recall method for the last three consecutive days (2 weekdays and 1 weekend day) were used to collect data regarding dietary intake. Based on the Dietary Guideline for Chinese Residents,^20^ the entire intake of cereals and beans was divided into three categories: insufficient (<40 g/d), sufficient (≥40 to ≤75 g/d), and excessive (>75 g/d). Similarly, mean and poultry intake was divided into three categories: insufficient (<50 g/d), sufficient (≥50 to ≤150 g/d), and excessive (>150 g/d). A physical activity questionnaire was used to collect information regarding physical activity variables, such as whether subjects exercised and sedentary time (watching TV, using computers, playing video games, reading, and doing homework) in leisure time. Body mass index (BMI) was calculated as weight in kilograms divided by height in meters squared (kg/m^2^).

Fasting glucose was measured by collecting morning fasting venous blood samples. Then, subjects without known diabetes were required to take a 75‐g oral glucose load and, 2 hours later, venous blood sample were collected to determine 2‐hour plasma glucose concentrations. According to the criteria proposed by World Health Organization, International Diabetes Federation 1999 and The American Diabetes Association on Diabetes Mellitus,^21^, ^22^, ^23^ impaired fasting glucose (IFG) was defined as fasting plasma glucose (FPG) ≥6.1 and <7.0 mM, and 2‐hour plasma glucose <7.8 mM. Impaired glucose tolerance (IGT) was defined as FPG <7.0 mM and 2‐hour plasma glucose ≥7.8 and <11.1 mM. Diabetes was defined as FPG ≥7.0 mM and/or 2‐hour plasma glucose ≥11.0 mM and/or a previous clinical diagnosis of diabetes.

### 2.4 Computation of genetic risk scores

A simple count method and a weighted method were used to create two genetic risk scores (GRSs). The weighted GRS was calculated on the basis of the 50 SNPs by using a previously described weighted method.^3^ Each SNP was weighted by β coefficients obtained from published meta‐analyses.^4^, ^7^, ^10^, ^11^, ^12^, ^16^, ^17^, ^24^, ^25^, ^26^, ^27^, ^28^, ^29^, ^30^, ^31^, ^32^, ^33^, ^34^, ^35^ (The original β coefficients can be found in the references listed in Table S2.) The weighted GRS was calculated by multiplying each β coefficients by the number of corresponding risk alleles and summing the products, then dividing the sum by twice the sum of the β coefficients and multiplying by 50. The non‐weighted GRS was calculated as the sum of the number of risk alleles for each SNP.

### 2.5 Statistical analysis

Chi‐squared and *t* tests were used for comparisons of proportions and means of baseline characteristics between male and female subjects.

In this study, general linear model (GLM) regression was used to test the relationship between FPG and each SNP, adjusting for covariates such as age, sex, education, economic status, exercise, sedentary time, smoking, drinking alcohol, meat and poultry intake, cereal and bean intake, and BMI. Logistic regression was used to estimate the odds ratios (ORs) for the risk of diabetes, IFG, and IGT after adjusting for the aforementioned covariates.

To determine the effects of genetic susceptibility to diabetes, the GRS was first treated as a continuous variable to test the relationship between genotype score and FPG, diabetes, IFG, and IGT by general linear or logistic regression. Then, according to quartiles of GRS, subjects were divided into four subgroups (Q1–Q4) and GLM regression was used to test the relationship between FPG and GRS after adjusting for covariates. Logistic regression was used to estimate ORs for the risk of diabetes, IFG, and IGT after adjusting for covariates. Moreover, to test for linear trends across quartiles of genotype score, the quartile medians were modeled as a continuous variable. Then, linear trend analysis was conducted between the GRS and FPG, diabetes, IFG, and IGT. In multivariate analyses, we adjusted for some established risk lifestyle factors and further adjusted for a family history of diabetes.

Statistical analyses were performed using SAS 9.4 (SAS Institute, Cary, North Carolina). Two‐tailed *P* < 0.05 was considered significant.

## 3 RESULTS

### 3.1 Subject characteristics

Subject characteristics are given in Table 1. In all, 2129 subjects (39.1% male, 60.9% female) were included in this study, with a mean (±SD) age of 49.9 ± 1.5 years. There were sex differences in education level, smoking, drinking, intake of meat and poultry, BMI, exercise, and the prevalence of diabetes.

**Table 1:** Subjects characteristics

	Total	Male	Female	*P*‐value
No. subjects	2129	833 (39.1)	1296 (60.9)	
Age (y)	49.9 ± 1.5	49.9 ± 1.5	50.0 ± 1.5	0.691
Education level				<0.001
Illiterate to primary school	745 (35.0)	187 (22.4)	558 (43.1)	
Junior middle school	922 (43.3)	414 (49.7)	508 (39.2)	
Senior high school or higher	462 (21.7)	232 (27.9)	230 (17.7)	
Family's economic level (Yuan/y per capita)				0.910
<20 000	1092 (51.3)	424 (50.9)	668 (51.5)	
20 000–40 000	804 (37.8)	320 (38.4)	484 (37.3)	
>40 000	155 (7.3)	61 (7.3)	94 (7.3)	
Missing	78 (3.7)			
Smoker				<0.001
No	1506 (70.7)	268 (32.2)	1238 (95.5)	
Yes	620 (29.1)	563 (67.6)	57 (4.4)	
Missing	3 (0.1)			
Drinker				<0.001
No	1423 (66.8)	313 (37.6)	1110 (85.6)	
Yes	704 (33.1)	519 (62.3)	185 (14.3)	
Missing	2 (0.1)			
Intake of cereals and beans^a^				0.580
Insufficient	1398 (65.7)	545 (65.4)	853 (65.8)	
Sufficient	171 (8.0)	62 (7.4)	109 (8.4)	
Excessive	42 (2.0)	20 (2.4)	22 (1.7)	
Missing	518 (24.3)			
Intake of meat and poultry^b^				<0.001
Insufficient	653 (30.7)	227 (27.3)	426 (32.9)	
Sufficient	365 (17.1)	117 (14.0)	248 (19.1)	
Excessive	593 (27.9)	283 (34.0)	310 (23.9)	
Missing	518 (24.3)			
BMI (kg/m^2^)	24.3 ± 3.4	24.0 ± 3.3	24.5 ± 3.4	<0.001
Exercise				0.027
No	1925 (90.4)	770 (92.4)	1155 (89.1)	
Yes	189 (8.9)	60 (7.2)	129 (10.0)	
Missing	15 (0.7)			
Sedentary time (h/d)	2.7 ± 1.5	2.7 ± 1.4	2.7 ± 1.5	0.420
FPG (mM)	5.3 ± 1.2	5.3 ± 1.2	5.3 ± 1.2	0.778
Diabetes				0.032
No	2000 (93.9)	771 (92.6)	1229 (94.8)	
Yes	129 (6.1)	62 (7.4)	67 (5.2)	
IFG				0.269
No	1876 (93.8)	729 (94.6)	1147 (93.3)	
Yes	124 (6.2)	42 (5.4)	82 (6.7)	
IGT				0.211
No	1885 (94.3)	733 (95.1)	1152 (93.7)	
Yes	115 (5.8)	38 (4.9)	77 (6.3)	

*Note*: Continuous variables are presented as the mean ± SD; categorical data are presented as n (%). *P*‐values were calculated using Chi‐squared test for categorical variables or *t* tests for continuous variables.

Abbreviations: BMI, body mass index; FPG, fasting plasma glucose; IFG, impaired fasting glucose; IGT, impaired glucose tolerance.

a Dietary intake of cereals and beans was divided into three categories: insufficient (<40 g/d), sufficient (from ≥40 to ≤75 g/d), and excessive (>75 g/d).

b Dietary intake of meat and poultry intake was also divided into three categories: insufficient (<50 g/d), sufficient (from ≥50 to ≤150 g/d), and excessive (>150 g/d).

### 3.2 Associations between individual SNPs and diabetes risk

After adjusting for age, sex, education, economic status, smoking, drinking, meat and poultry intake, cereal and bean intake, exercise, sedentary time, and BMI, significant associations were observed between diabetes and rs10946398, rs2028299, rs4858889, and rs7403531; between IGT and rs10886471, rs2191349, and rs9470794; between IFG and rs10842994; and between FPG and rs10830963, rs2028299, rs516946 and rs7403531 (Table 2).

**Table 2:** Associations between single nucleotide polymorphisms and diabetes risk in the Chinese population^a^

SNP	Diabetes^b^	IGT^b^	IFG^b^	FPG^c^
rs10401969	0.85 (0.52, 1.38)	0.97 (0.60, 1.58)	1.20 (0.78, 1.86)	−0.000 (−0.131, 0.131)
rs10830963	1.21 (0.92, 1.59)	0.83 (0.62, 1.11)	1.12 (0.84, 1.48)	0.094 (0.016, 0.171)*
rs10842994	1.41 (0.97, 2.03)	1.16 (0.81, 1.67)	0.66 (0.48, 0.90)*	0.074 (−0.024, 0.171)
rs10886471	1.37 (0.97, 1.94)	1.68 (1.14, 2.50)*	1.12 (0.79, 1.58)	0.152 (0.057, 0.247)
rs10906115	1.01 (0.76, 1.34)	1.17 (0.87, 1.57)	0.88 (0.67, 1.17)	0.009 (−0.070, 0.089)
rs10946398	1.59 (1.21, 2.08)*	1.02 (0.77, 1.36)	1.14 (0.86, 1.50)	0.060 (−0.018, 0.138)
rs11257655	1.10 (0.83, 1.45)	1.10 (0.82, 1.46)	0.88 (0.66, 1.16)	0.035 (−0.044, 0.113)
rs11634397	1.17 (0.78, 1.75)	1.18 (0.77, 1.81)	1.15 (0.75, 1.76)	0.041 (−0.083, 0.164)
rs12454712	1.13 (0.87, 1.46)	0.89 (0.68, 1.17)	0.79 (0.61, 1.04)	0.003 (−0.073, 0.079)
rs12970134	0.92 (0.64, 1.32)	1.17 (0.83, 1.66)	1.00 (0.70, 1.43)	−0.031 (−0.130, 0.067)
rs13266634	0.87 (0.66, 1.15)	0.95 (0.72, 1.27)	0.87 (0.66, 1.15)	−0.013 (−0.090, 0.065)
rs1470579	1.03 (0.76, 1.39)	1.00 (0.73, 1.37)	1.03 (0.76, 1.40)	0.004 (−0.083, 0.090)
rs1535500	1.05 (0.81, 1.37)	0.96 (0.73, 1.26)	0.93 (0.71, 1.21)	0.022 (−0.053, 0.097)
rs1552224	1.17 (0.73, 1.87)	1.20 (0.72, 2.01)	0.79 (0.51, 1.22)	0.013 (−0.123, 0.150)
rs1558902	0.87 (0.58, 1.30)	0.94 (0.62, 1.43)	1.23 (0.85, 1.79)	−0.033 (−0.148, 0.081)
rs16861329	0.92 (0.67, 1.27)	1.17 (0.85, 1.61)	1.01 (0.73, 1.40)	−0.034 (−0.124, 0.055)
rs17584499	0.87 (0.55, 1.37)	1.35 (0.91, 2.00)	1.24 (0.83, 1.84)	0.036 (−0.085, 0.157)
rs2028299	1.51 (1.13, 2.01)*	1.32 (0.96, 1.80)	0.87 (0.62, 1.23)	0.098 (0.006, 0.190)*
rs2191349	0.95 (0.72, 1.24)	0.73 (0.56, 0.96)*	1.25 (0.94, 1.66)	0.049 (−0.029, 0.128)
rs243021	0.96 (0.72, 1.26)	1.07 (0.80, 1.44)	1.09 (0.81, 1.46)	0.019 (−0.061, 0.099)
rs2796441	1.11 (0.85, 1.44)	1.02 (0.77, 1.36)	0.87 (0.66, 1.15)	0.030 (−0.047, 0.107)
rs2943641	1.15 (0.70, 1.91)	0.66 (0.43, 1.03)	1.08 (0.65, 1.79)	0.006 (−0.133, 0.145)
rs340874	0.92 (0.70, 1.20)	1.06 (0.80, 1.40)	0.90 (0.68, 1.19)	−0.011 (−0.090, 0.067)
rs3794991	0.84 (0.49, 1.46)	0.69 (0.36, 1.30)	0.72 (0.39, 1.33)	0.000 (−0.152, 0.153)
rs3923113	1.00 (0.69, 1.46)	0.95 (0.65, 1.39)	1.16 (0.78, 1.74)	0.014 (−0.092, 0.120)
rs4430796	1.05 (0.78, 1.40)	1.15 (0.85, 1.55)	0.82 (0.60, 1.12)	0.006 (−0.079, 0.090)
rs459193	0.94 (0.72, 1.22)	1.10 (0.84, 1.45)	1.00 (0.77, 1.31)	0.032 (−0.044, 0.107)
rs4607103	0.90 (0.69, 1.18)	1.12 (0.84, 1.48)	0.91 (0.70, 1.20)	−0.064 (−0.140, 0.012)
rs4607517	0.82 (0.59, 1.14)	1.10 (0.80, 1.52)	1.03 (0.75, 1.42)	−0.030 (−0.121, 0.061)
rs4858889	0.68 (0.48, 0.96)*	0.90 (0.61, 1.33)	0.75 (0.53, 1.07)	−0.019 (−0.126, 0.089)
rs5015480	0.99 (0.70, 1.40)	0.97 (0.68, 1.38)	1.18 (0.84, 1.66)	−0.006 (−0.105, 0.092)
rs516946	1.61 (0.99, 2.60)	1.05 (0.68, 1.60)	0.87 (0.58, 1.30)	0.146 (0.029, 0.263)*
rs5215	1.06 (0.80, 1.40)	1.29 (0.97, 1.71)	1.10 (0.83, 1.45)	−0.004 (−0.082, 0.074)
rs6815464	1.06 (0.80, 1.39)	0.96 (0.73, 1.27)	1.19 (0.90, 1.58)	0.010 (−0.068, 0.088)
rs7041847	1.14 (0.87, 1.49)	1.24 (0.93, 1.64)	1.05 (0.79, 1.38)	0.034 (−0.044, 0.111)
rs7172432	1.08 (0.82, 1.42)	1.12 (0.84, 1.49)	1.34 (0.99, 1.79)	0.065 (−0.014, 0.144)
rs7178572	1.11 (0.85, 1.46)	1.08 (0.82, 1.43)	1.30 (0.98, 1.72)	0.064 (−0.016, 0.144)
rs7202877	1.18 (0.84, 1.65)	1.26 (0.88, 1.82)	0.97 (0.69, 1.36)	0.059 (−0.037, 0.156)
rs7403531	1.37 (1.04, 1.80)*	0.75 (0.55, 1.02)	1.16 (0.88, 1.54)	0.127 (0.045, 0.209)*
rs7593730	1.05 (0.73, 1.50)	0.99 (0.69, 1.42)	0.76 (0.55, 1.06)	−0.041 (−0.137, 0.056)
rs7612463	1.02 (0.73, 1.41)	1.07 (0.76, 1.52)	1.11 (0.79, 1.57)	−0.015 (−0.106, 0.076)
rs780094	0.91 (0.69, 1.19)	0.93 (0.70, 1.23)	1.11 (0.84, 1.46)	0.008 (−0.067, 0.083)
rs7961581	0.81 (0.57, 1.14)	0.71 (0.50, 1.02)	1.19 (0.87, 1.63)	0.023 (−0.068, 0.114)
rs8050136	0.95 (0.64, 1.42)	0.95 (0.62, 1.44)	1.33 (0.91, 1.93)	−0.023 (−0.137, 0.091)
rs8090011	0.83 (0.63, 1.09)	1.07 (0.79, 1.44)	1.00 (0.74, 1.36)	−0.059 (−0.146, 0.027)
rs831571	0.81 (0.61, 1.08)	0.79 (0.59, 1.05)	0.97 (0.73, 1.28)	0.033 (−0.046, 0.111)
rs864745	1.05 (0.76, 1.45)	0.91 (0.66, 1.25)	0.98 (0.71, 1.36)	0.016 (−0.075, 0.106)
rs896854	0.94 (0.71, 1.24)	0.83 (0.61, 1.12)	1.08 (0.81, 1.43)	−0.028 (−0.108, 0.051)
rs9470794	1.16 (0.87, 1.55)	1.71 (1.24, 2.38)*	0.95 (0.72, 1.27)	0.057 (−0.022, 0.136)
rs972283	0.96 (0.72, 1.30)	0.90 (0.67, 1.22)	1.03 (0.76, 1.39)	0.017 (−0.068, 0.101)

Abbreviations: IFG, impaired fasting glucose; IGT, impaired glucose tolerance; SNP, single nucleotide polymorphism.

a After adjustment for age, sex, education, economic status, smoking, drinking, meat and poultry intake, cereal and bean intake, exercise, sedentary time, and body mass index.

b Data are presented as odds ratios with 95% confidence intervals in parentheses.

c Data are presented as β coefficients with 95% confidence intervals for increments of fasting plasma glucose (FPG).

* *P* < 0.05.

### 3.3 Association of GRS or weighted GRS with FPG

The median GRS was 20.00, whereas the median weighted GRS was 19.33. As a continuous variable, with a 1‐point increase in the GRS, FPG increased by 0.042 mM (*P <* 0.001). Further adjustment for covariates did not change the association between GRS and FPG (*P <* 0.001). Significant associations were found among all total subjects and female subjects. The results for the weighted GRS were similar. When the GRS and weighted GRS were divided into quartiles, linear trend analysis indicated that FPG increased with GRS (*P*_trend_ < 0.001) and weighted GRS (*P*_trend_ = 0.002) after adjusting for covariates. The linear relationship was significant among female subjects. Significant relationships for GRS or weighted GRS and diabetes risk were not found among male subjects (Table 3).

**Table 3:** Association of the genetic risk score (GRS) and weighted GRS with fasting plasma glucose in a Chinese population

Characteristic	Continuous score (*P‐*value)	Score quartiles (*P‐*value)				*P*_trend_
		Q1	Q2	Q3	Q4	
GRS						
Unadjusted	0.042 (<0.001)	1.00	0.101 (0.205)	0.155 (0.028)	0.210 (0.003)	0.002
Adjusted^a^	0.045 (<0.001)	1.00	0.103 (0.204)	0.170 (0.017)	0.233 (0.001)	<0.001
Male sex						
Unadjusted	0.027 (0.152)	1.00	0.148 (0.225)	0.060 (0.583)	0.113 (0.319)	0.370
Adjusted^a^	0.036 (0.062)	1.00	0.177 (0.148)	0.117 (0.288)	0.152 (0.180)	0.189
Female sex						
Unadjusted	0.051 (<0.001)	1.00	0.053 (0.569)	0.301 (0.003)	0.270 (0.003)	<0.001
Adjusted^a^	0.052 (<0.001)	1.00	0.044 (0.647)	0.291 (0.005)	0.280 (0.003)	<0.001
Weighted GRS						
Unadjusted	0.040 (0.002)	1.00	−0.030 (0.687)	0.053 (0.476)	0.189 (0.011)	0.006
Adjusted^a^	0.044 (<0.001)	1.00	0.003 (0.964)	0.091 (0.226)	0.221 (0.003)	0.002
Male sex						
Unadjusted	0.022 (0.279)	1.00	−0.178 (0.125)	−0.135 (0.244)	0.067 (0.564)	0.483
Adjusted^a^	0.029 (0.156)	1.00	−0.190 (0.103)	−0.084 (0.470)	0.100 (0.391)	0.261
Female sex						
Unadjusted	0.050 (0.002)	1.00	0.051 (0.598)	0.154 (0.113)	0.293 (0.003)	0.001
Adjusted^a^	0.053 (0.002)	1.00	0.093 (0.350)	0.177 (0.074)	0.315 (0.002)	0.001

*Note*: Data are presented as β coefficients for increments of fasting plasma glucose.

a Adjusted for age, sex, education, economic status, smoking, drinking, meat and poultry intake, cereal and bean intake, exercise, sedentary time, and body mass index.

### 3.4 Association of GRS or weighted GRS with diabetes risk

After adjusting for covariates, the ORs (95% confidence intervals [CIs]) of diabetes associated with a 1‐point increase in GRS were 1.09 (1.00, 1.19) among all subjects and 1.14 (1.00, 1.31) among male subjects. After adjusting for covariates, the ORs (95% CIs) of diabetes associated with a 1‐point increase of weighted GRS were 1.12 (1.03, 1.22) among all subjects and 1.18 (1.03, 1.35) among male subjects. After adjusting for covariates, compared with subjects in the lowest quartile of weighted GRS (Q1), those in Q4 of the GRS had a higher diabetes risk, with ORs (95% CIs) of 1.88 (1.12, 3.13) and 2.18 (1.01, 4.71) among all and male subjects, respectively.

The linear trend analysis indicated that diabetes risk increased with weighted GRS among all subjects (*P*_trend_ = 0.007) and among male subjects (*P*_trend_ = 0.017) after adjusting for covariates. No significant association between GRS or weighted GRS and IFG or IGT was found whether covariates were adjustment for or not (Table 4).

**Table 4:** Association of the genetic risk score (GRS) and weighted GRS with diabetes risk in a Chinese population

Characteristic	Continuous score	Score quartiles				*P*_trend_
		Q1	Q2	Q3	Q4	
GRS						
Diabetes						
Unadjusted	1.07 (0.99, 1.17)	1.00	1.25 (0.72, 2.17)	1.10 (0.67, 1.82)	1.42 (0.88, 2.30)	0.193
Adjusted^a^	1.09 (1.00, 1.19)	1.00	1.32 (0.75, 2.34)	1.16 (0.69, 1.97)	1.64 (0.99, 2.71)	0.074
Male sex						
Unadjusted	1.10 (0.97, 1.24)	1.00	1.64 (0.77, 3.50)	1.15 (0.55, 2.41)	1.39 (0.67, 2.90)	0.478
Adjusted^a^	1.14 (1.00, 1.31)	1.00	2.03 (0.89, 4.63)	1.45 (0.65, 3.24)	1.86 (0.84, 4.10)	0.163
Female sex						
Unadjusted	1.06 (0.95, 1.19)	1.00	0.76 (0.36, 1.62)	1.33 (0.65, 2.71)	1.45 (0.76, 2.77)	0.161
Adjusted^a^	1.06 (0.94, 1.19)	1.00	0.73 (0.34, 1.58)	1.20 (0.57, 2.52)	1.47 (0.76, 2.84)	0.165
IFG						
Unadjusted	1.02 (0.94, 1.11)	1.00	0.97 (0.55, 1.69)	0.94 (0.58, 1.54)	1.11 (0.68, 1.80)	0.729
Adjusted^a^	1.02 (0.94, 1.12)	1.00	0.95 (0.53, 1.69)	0.87 (0.52, 1.44)	1.14 (0.69, 1.87)	0.709
Male sex						
Unadjusted	0.95 (0.82, 1.09)	1.00	0.87 (0.35, 2.12)	0.70 (0.31, 1.62)	0.81 (0.35, 1.87)	0.510
Adjusted^a^	0.94 (0.81, 1.09)	1.00	0.77 (0.29, 2.00)	0.65 (0.28, 1.53)	0.80 (0.34, 1.89)	0.484
Female sex						
Unadjusted	1.06 (0.95, 1.17)	1.00	1.03 (0.55, 1.94)	1.16 (0.59, 2.27)	1.30 (0.71, 2.37)	0.361
Adjusted^a^	1.05 (0.95, 1.17)	1.00	0.92 (0.47, 1.78)	1.04 (0.52, 2.10)	1.30 (0.69, 2.42)	0.374
IGT						
Unadjusted	1.04 (0.95, 1.13)	1.00	0.83 (0.45, 1.50)	1.04 (0.63, 1.70)	1.04 (0.63, 1.72)	0.809
Adjusted^a^	1.04 (0.96, 1.14)	1.00	0.83 (0.45, 1.52)	1.05 (0.64, 1.73)	1.09 (0.65, 1.81)	0.676
Male sex						
Unadjusted	0.97 (0.84, 1.13)	1.00	0.53 (0.19, 1.51)	0.70 (0.31, 1.62)	0.72 (0.31, 1.71)	0.421
Adjusted^a^	0.98 (0.84, 1.14)	1.00	0.49 (0.17, 1.42)	0.67 (0.29, 1.59)	0.74 (0.31, 1.80)	0.448
Female sex						
Unadjusted	1.07 (0.96, 1.19)	1.00	0.96 (0.49, 1.89)	1.55 (0.80, 2.99)	1.26 (0.67, 2.38)	0.345
Adjusted^a^	1.08 (0.97, 1.21)	1.00	0.99 (0.50, 1.96)	1.56 (0.79, 3.05)	1.38 (0.72, 2.63)	0.232
Weighted GRS						
Diabetes						
Unadjusted	1.09 (1.01, 1.19)	1.00	0.92 (0.53, 1.60)	1.15 (0.68, 1.94)	1.58 (0.97, 2.59)	0.038
Adjusted^a^	1.12 (1.03, 1.22)	1.00	0.96 (0.54, 1.70)	1.29 (0.75, 2.22)	1.88 (1.12, 3.13)	0.007
Male sex						
Unadjusted	1.13 (1.00, 1.28)	1.00	0.84 (0.37, 1.92)	1.33 (0.63, 2.81)	1.68 (0.82, 3.46)	0.083
Adjusted^a^	1.18 (1.03, 1.35)	1.00	0.81 (0.33, 1.94)	1.60 (0.71, 3.59)	2.18 (1.01, 4.71)	0.017
Female sex						
Unadjusted	1.08 (0.96, 1.21)	1.00	0.68 (0.31, 1.48)	1.13 (0.57, 2.26)	1.40 (0.72, 2.72)	0.173
Adjusted^a^	1.09 (0.97, 1.22)	1.00	0.70 (0.31, 1.58)	1.19 (0.59, 2.41)	1.54 (0.78, 3.06)	0.114
IFG						
Unadjusted	1.04 (0.96, 1.14)	1.00	0.73 (0.42, 1.26)	1.01 (0.60, 1.68)	1.32 (0.81, 2.16)	0.150
Adjusted^a^	1.05 (0.96, 1.14)	1.00	0.81 (0.46, 1.43)	1.07 (0.63, 1.82)	1.35 (0.81, 2.26)	0.161
Male sex						
Unadjusted	1.00 (0.85, 1.16)	1.00	0.56 (0.22, 1.46)	0.93 (0.40, 2.15)	1.05 (0.46, 2.39)	0.720
Adjusted^a^	1.01 (0.86, 1.19)	1.00	0.56 (0.21, 1.50)	1.07 (0.44, 2.61)	1.15 (0.48, 2.76)	0.516
Female sex						
Unadjusted	1.06 (0.96, 1.17)	1.00	0.82 (0.41, 1.62)	1.12 (0.59, 2.13)	1.43 (0.78, 2.65)	0.162
Adjusted^a^	1.05 (0.94, 1.17)	1.00	0.90 (0.45, 1.82)	1.09 (0.56, 2.13)	1.36 (0.71, 2.60)	0.292
IGT						
Unadjusted	1.03 (0.94, 1.13)	1.00	0.96 (0.56, 1.65)	1.24 (0.74, 2.07)	0.95 (0.55, 1.65)	0.920
Adjusted^a^	1.04 (0.95, 1.13)	1.00	1.00 (0.58, 1.73)	1.26 (0.75, 2.13)	1.00 (0.57, 1.74)	0.815
Male sex						
Unadjusted	1.00 (0.85, 1.18)	1.00	0.89 (0.35, 2.23)	1.02 (0.41, 2.50)	0.94 (0.37, 2.36)	0.950
Adjusted^a^	1.02 (0.86, 1.21)	1.00	0.77 (0.30, 2.00)	1.17 (0.46, 2.97)	0.98 (0.38, 2.54)	0.864
Female sex						
Unadjusted	1.04 (0.93, 1.15)	1.00	0.98 (0.51, 1.89)	1.12 (0.59, 2.13)	0.96 (0.50, 1.87)	0.995
Adjusted^a^	1.05 (0.95, 1.18)	1.00	1.07 (0.55, 2.09)	1.14 (0.59, 2.19)	1.10 (0.56, 2.17)	0.744

*Note*: Data are presented as odds ratios (95% confidence interval) for the risk of diabetes, impaired fasting glucose (IFG), and impaired glucose tolerance (IGT).

a Adjusted for age, sex, education, economic status, smoking, drinking, meat and poultry intake, cereal and bean intake, exercise, sedentary time, and body mass index.

## 4 DISCUSSION

Diabetes mellitus is a group of metabolic diseases characterized by hyperglycemia. Diabetes is caused by a progressive loss of β‐cell insulin secretion frequently against a background of insulin resistance, or autoimmune β‐cell destruction, usually leading to absolute insulin deficiency.^22^, ^23^ Among the 50 SNPs included in this study, most loci exerted their primary effects on disease risk through deficient insulin secretion, some loci were related to insulin resistance or insulin sensibility, and some loci may be the adapter or receptor that can indirectly affect insulin sensitivity or increase diabetes susceptibility.^8^, ^10^, ^28^, ^32^, ^36^, ^37^, ^38^, ^39^, ^40^, ^41^, ^42^, ^43^, ^44^

Among the susceptibility loci examined herein, we confirmed significant evidence for an association with diabetes risk for 10 loci in the Chinese population in the following genes: *cyclindependentkinase 5 regulatory subunit associated protein 1 (CDKAL1)* (rs10946398), *adaptorrelated protein complex 3 subunit sigma 2 (AP3S2)* (rs2028299), *SREBF chaperone (SCAP)* (rs4858889), RAS guanyl releasing protein 1 (*RASGRP1)* (rs7403531), Gprotein‐coupled receptor kinase 5 (*GRK5)* (rs10886471), *diacylglycerol kinase beta/transmembrane protein 195 (DGKB/TMEM195)* (rs2191349), zinc finger AN1‐type containing 3 (*ZFAND3*) (rs9470794), kelchdomain containing 5 (*KLHDC5*) (rs10842994), *melatonin receptor 1B (MTNR1B)* (rs10830963), and *ankyrin1 (ANK1)* (rs516946). Previous studies have identified an association between diabetes risk and SNPs for *CDKAL1* in European Americans, African Americans, UK samples, Indians, Korean, and Chinese,^33^, ^45^, ^46^, ^47^, ^48^, ^49^ making *CDKAL1* one of the most highly replicated genes identified. A study in a Chinese Han population reported an OR of 1.47 (95% CI 1.25‐1.73) for the association of rs10946398 in *CDKAL1* with diabetes,^48^ which is similar to the findings of the present study (OR 1.59; 95% CI 1.21‐2.08). The direction and magnitude of the association of rs4858889 in *SCAP* with diabetes in the present study (OR 0.68; 95% CI 0.48‐0.96) are consistent with the findings in a previous study in south Asians (OR 0.84; 95% CI 0.77‐0.91).^17^ In contrast, another Chinese study did not find a significant association between rs4858889 and diabetes;^50^ however, that study was a case‐control study and the sample was enrolled from hospitals, hence the study cohort may not be representative of the general population. In addition, in the present study we adjusted for more covariates in addition to age, sex, and BMI. The subjects in the present study were born in the early 1960s, which maybe another reason for the apparent discrepancy between studies. In the early 1960s, China had just experienced severe famine, and some studies have found that experiencing famine or malnutrition in early life may increase susceptibility to diabetes.^51^, ^52^

The findings in the present study for other genes are consistent with previous studies for *RASGRP1* and *GRK5* in Chinese,^10^ *ZFAND3* in East Asians,^12^ *MTNR1B* in Europeans, Koreans, and Chinese,^53^, ^54^, ^55^ and *ANK1* in Chinese.^56^ In this study, we observed a significant association of rs2028299 near *AP3S2* with diabetes and FPG. This SNP was identified as a susceptibility locus for type 2 diabetes in a GWAS in South Asian populations, a Japanese population and a northern Chinese Han population.^28^, ^57^, ^58^ We observed that rs2191349 reduced IGT risk, which was the same direction as reported in a Korean study.^59^ However, in a population‐based prospective cohort study from northern Sweden, rs2191349 was associated with elevated IFG risk.^60^ In the present study we found that rs10842994 was associated with a reduced risk of IFG, but a study conducted in a Japanese population examined the association of rs10842994 near *KLHDC5* with susceptibility to diabetes.^34^ These two SNPs need to be evaluated further in a larger sample of the Chinese or in different populations. The direction of the effect of most SNPs in the present study was the same as reported in previous studies. Therefore, it is important to evaluate the effect of each locus in different ethnic groups or a larger sample of the Chinese population. Insufficient sample size may be a principal explanation for the discrepancies between the present study and previous studies.

In the present study we computed a weighted GRS by using the reported β coefficients. The GRS and weighted GRS were expanded by inclusion of 50 SNPs. The GRS and weighted GRS were significantly associated with an increase in FPG after adjusting for covariates. Each additional GRS or weighted GRS, corresponding to one risk allele, was associated with a 9% and 12% increase, respectively, in the odds of developing diabetes among subjects. The association between genetic susceptibility and the risk of diabetes reported here is consistent with the findings of the other studies. A study among African American populations counted the β‐cell dysfunction (BCD) GRS and/or insulin resistance (IR) GRS separately, and reported that the BCD GRS and combined BCD/IR GRS were significantly associated with increased type 2 diabetes risk.^61^ Two studies conducted among European American nurses and health professionals also found association between GRS or weighted GRS and the risk of diabetes.^45^, ^62^ In a follow‐up study among Finnish men, a non‐weighted GRS for type 2 diabetes and a weighted GRS for FPG and IR were associated with incident type 2 diabetes.^63^ Similar results have been found in Asian populations, with Korean adults with a higher GRS having higher type 2 diabetes risk^64^ and a tendency for impaired insulin secretion among a Chinese Han population.^65^ In the present study, significant results were found between GRS or weighted GRS and FPG among female subjects. Significant results were also found between GRS or weighted GRS and diabetes among male subjects. However, no other significant results were found. Previous studies have found an association between GRS and diabetes risk in both men and women.^45^, ^62^ A study conducted in Finland only explored the association between diabetes risk and GRS in men.^63^ Other Asian population studies did not consider sex differences.^64^, ^65^ Sex differences may need to be confirmed in future studies.

The present study has several strengths. First, this study examined the genetic susceptibility in relation to the risk of diabetes in adults in a Chinese population that came from a nationally representative cross‐sectional study. In addition, this study explored genetic susceptibility associated with diabetes by creating a GRS and a weighted GRS including 50 SNPs. Furthermore, the association between each SNP and the risk of diabetes was analyzed, and a range of behavioral factors, including smoking, drinking, and dietary and exercise factors that had been reported as risk factors for diabetes, were considered.

The present study also has some limitations. First, although we adjusted for some covariates, including dietary and lifestyle factors, quantitative indices for alcohol intake and exercise were not available in this study, which may have reduced the power of the study to explore any associations. Second, the SNPs included in this study did not cover all SNPs identified as diabetes risk loci, and only subjects born in early 1960s were analyzed.

In conclusion, we confirmed the association of 10 SNPs with diabetes risk, and observed associations of GRS or weighted GRS with FPG among Chinese females and with diabetes among Chinese males born in the early 1960s. We also found a linear trend between genetic susceptibility and diabetes or FPG.

## DISCLOSURE

None declared.

## Supporting information

Table S1 Hardy‐Weinberg equilibrium test
Table S2. Characteristics of 50 established single nucleotide polymorphisms for diabetes
