# Supplementary material for: Effects of variants of 50 genes on diabetes risk among the Chinese population born in the early 1960s
Source: J Diabetes. 2019 Apr 25;11(11):857–68. doi: 10.1111/1753-0407.12922 (PMC6850447; doi:10.1111/1753-0407.12922)
Supplement: Supplementary file 1 — Table S1 Hardy‐Weinberg equilibrium test Table S2. Characteristics of 50 established single nucleotide polymorphisms for diabetes [file JDB-11-857-s001.docx]

**Supplementary Table 1. Hardy-Weinberg equilibrium test**

| **SNP** | **Chromosome** | **Loci** | **Allele** | **Genotype** | **Genotype frequency** | ***P* Value** |
| --- | --- | --- | --- | --- | --- | --- |
| rs340874 | 1 | PROX1 | C/T | CC/CT/TT | 309/939/699 | 0.830 |
| rs243021 | 2 | BCL11A | G/A | AA/GA/GG | 919/850/200 | 0.867 |
| rs2943641 | 2 | IRS1 | C/T | CC/CT/TT | 1709/275/14 | 0.423 |
| rs3923113 | 2 | GRB14 | C/A | AA/CA/CC | 1463/463/46 | 0.196 |
| rs7593730 | 2 | RBMS1, ITGB6 | C/T | CC/CT/TT | 1397/541/56 | 0.678 |
| rs780094 | 2 | GCKR | T/C | CC/TC/TT | 452/962/477 | 0.443 |
| rs1470579 | 3 | IGF2BP2 | C/A | AA/CA/CC | 1117/751/120 | 0.675 |
| rs16861329 | 3 | ST6GAL1 | T/C | CC/TC/TT | 1158/696/105 | 0.975 |
| rs4607103 | 3 | ADAMTS9 | C/T | CC/CT/TT | 794/900/289 | 0.191 |
| rs4858889 | 3 | SCAP | G/A | AA/GA/GG | 1417/491/38 | 0.548 |
| rs7612463 | 3 | UBE2E2 | C/A | AA/CA/CC | 80/609/1288 | 0.454 |
| rs831571 | 3 | PSMD6 | C/T | CC/CT/TT | 794/935/261 | 0.583 |
| rs6815464 | 4 | MAEA | C/G | CC/CG/GG | 674/981/315 | 0.181 |
| rs459193 | 5 | ANKRD55 | G/A | AA/GA/GG | 465/1000/510 | 0.558 |
| rs10946398 | 6 | CDKAL1 | C/A | AA/CA/CC | 665/1016/296 | 0.004 |
| rs1535500 | 6 | KCNK16 | G/T | GG/0/TT | 578/972/430 | 0.573 |
| rs9470794 | 6 | ZFAND3 | C/T | CC/CT/TT | 205/839/942 | 0.369 |
| rs2191349 | 7 | DGKB, TMEM195 | G/T | GG/0/TT | 261/853/824 | 0.089 |
| rs4607517 | 7 | GCK | G/A | AA/GA/GG | 102/661/1202 | 0.371 |
| rs864745 | 7 | JAZF1 | C/T | CC/CT/TT | 91/752/1115 | 0.011 |
| rs972283 | 7 | KLF14 | G/A | AA/GA/GG | 147/800/1025 | 0.595 |
| rs13266634 | 8 | SLC30A8 | T/C | CC/TC/TT | 592/1034/347 | 0.004 |
| rs516946 | 8 | ANK1 | T/C | CC/TC/TT | 1533/444/19 | 0.033 |
| rs896854 | 8 | TP53INP1 | C/T | CC/CT/TT | 901/859/220 | 0.479 |
| rs10811661 | 9 | CDKN2A, CDKN2B | T/C | CC/TC/TT | 365/1014/517 | <0.001 |
| rs13292136 | 9 | CHCHD9 | T/C | CC/TC/TT | 1997/6/17 | <0.001 |
| rs17584499 | 9 | PTPRD | C/T | CC/CT/TT | 1604/366/24 | 0.546 |
| rs2796441 | 9 | TLE1 | A/G | AA/AG/GG | 699/931/311 | 0.973 |
| rs7041847 | 9 | GLIS3 | A/G | AA/AG/GG | 408/1035/520 | 0.010 |
| rs10886471 | 10 | GRK5 | T/C | CC/TC/TT | 1149/665/83 | 0.282 |
| rs10906115 | 10 | CDC123, CAMK1D | A/G | AA/AG/GG | 782/963/243 | 0.042 |
| rs11257655 | 10 | CDC123 | C/T | CC/CT/TT | 337/979/635 | 0.223 |
| rs12571751 | 10 | ZMIZ1 | G/A | AA/GA/GG | 1605/79/292 | <.001 |
| rs1802295 | 10 | VPS26A | T/C | CC/TC/TT | 1164/493/397 | <.001 |
| rs5015480 | 10 | HHEX | C/T | CC/CT/TT | 60/593/1295 | 0.427 |
| rs10830963 | 11 | MTNR1B | C/G | CC/CG/GG | 635/1024/319 | 0.005 |
| rs1552224 | 11 | CENTD2 | C/A | AA/CA/CC | 1683/314/11 | 0.374 |
| rs2237892 | 11 | KCNQ1 | T/C | CC/TC/TT | 848/975/199 | <0.001 |
| rs231362 | 11 | KCNQ1 | G/A | AA/GA/GG | 34/25/1901 | <0.001 |
| rs5215 | 11 | KCNJ11 | C/T | CC/CT/TT | 291/993/711 | 0.063 |
| rs10842994 | 12 | KLHDC5 | C/T | CC/CT/TT | 1313/647/57 | 0.032 |
| rs1531343 | 12 | HMGA2 | G/C | CC/GC/GG | 13/21/1967 | <0.001 |
| rs7961581 | 12 | TSPAN8, LGR5 | C/T | CC/CT/TT | 91/688/1208 | 0.579 |
| rs1359790 | 13 | SPRY2 | G/A | AA/GA/GG | 35/216/1731 | <0.001 |
| rs11634397 | 15 | ZFAND6 | A/G | AA/AG/GG | 1626/331/28 | 0.020 |
| rs2028299 | 15 | AP3S2 | C/A | AA/CA/CC | 1278/655/80 | 0.731 |
| rs7172432 | 15 | C2CD4A, C2CD4B | G/A | AA/GA/GG | 782/942/259 | 0.347 |
| rs7178572 | 15 | HMG20A | A/G | AA/AG/GG | 780/960/242 | 0.042 |
| rs7403531 | 15 | RASGRP1 | T/C | CC/TC/TT | 777/815/229 | 0.501 |
| rs1558902 | 16 | FTO | A/T | AA/AT/TT | 38/405/1511 | 0.078 |
| rs7202877 | 16 | BCAR1 | G/T | GG/0/TT | 70/632/1292 | 0.498 |
| rs8050136 | 16 | FTO | C/A | AA/CA/CC | 32/420/1544 | 0.576 |
| rs4430796 | 17 | HNF1B | A/G | AA/AG/GG | 989/802/161 | 0.929 |
| rs12454712 | 18 | BCL2 | C/T | CC/CT/TT | 385/996/609 | 0.537 |
| rs12970134 | 18 | MC4R | G/A | AA/GA/GG | 62/535/1319 | 0.392 |
| rs8090011 | 18 | LAMA1 | C/G | CC/CG/GG | 158/723/981 | 0.132 |
| rs10401969 | 19 | CILP2 | C/T | CC/CT/TT | 18/303/1699 | 0.274 |
| rs3786897 | 19 | PEPD | G/A | AA/GA/GG | 489/1075/383 | <0.001 |
| rs3794991 | 19 | GATAD2A | T/C | CC/TC/TT | 1713/219/14 | 0.019 |
| rs4812829 | 20 | HNF4A | G/A | AA/GA/GG | 573/779/636 | <0.001 |
| rs6017317 | 20 | HNF4A | G/T | GG/0/TT | 223/906/565 | <0.001 |

Supplementary Table 2. Characteristics of 50 established SNPs for diabetes

| **SNP** | **CHR** | **Loci** | **Risk allele** | **Other allele** | βbeta | **Reference** |
| --- | --- | --- | --- | --- | --- | --- |
| rs340874 | 1 | PROX1 | C | T | 0.068 | Morris AP et al, 2012(35) |
| rs780094 | 2 | GCKR | C | T | 0.110 | Dupuis J et al, 2010(37) |
| rs243021 | 2 | BCL11A | A | G | 0.080 | Voight BF et al, 2010(40) |
| rs7593730 | 2 | RBMS1, ITGB6 | C | T | 0.100 | Qi L et al, 2010(25) |
| rs3923113 | 2 | GRB14 | A | C | 0.090 | Kooner JS et al, 2011(31) |
| rs2943641 | 2 | IRS1 | C | T | 0.170 | Rung, J et al, 2009(16) |
| rs1470579 | 3 | IGF2BP2 | C | A | 0.113 | Morris AP et al, 2012(35) |
| rs831571 | 3 | PSMD6 | t | c | -0.090 | Cho YS et al, 2011(36) |
| rs4607103 | 3 | ADAMTS9 | C | T | 0.090 | Zeggini E et al, 2008(7) |
| rs7612463 | 3 | UBE2E2 | C | A | 0.100 | Mahajan A et al, 2014(32) |
| rs16861329 | 3 | ST6GAL1 | T | C | -0.150 | Wang H et al, 2017(33) |
| rs6815464 | 4 | MAEA | c | g | 0.101 | Cho YS et al, 2011(36) |
| rs459193 | 5 | ANKRD55 | G | A | 0.077 | Morris AP et al, 2012(35) |
| rs9470794 | 6 | ZFAND3 | t | c | -0.097 | Cho YS et al, 2011(36) |
| rs10946398 | 6 | CDKAL1 | C | A | 0.110 | Zeggini E et al, 2007(38) |
| rs1535500 | 6 | KCNK16 | T | G | 0.120 | Mahajan A et al, 2014(32) |
| rs4607517 | 7 | GCK | A | G | 0.060 | Dupuis J et al, 2010(37) |
| rs972283 | 7 | KLF14 | G | A | 0.070 | Voight BF et al, 2010(40) |
| rs2191349 | 7 | DGKB, TMEM195 | T | G | 0.060 | Dupuis J et al, 2010(37) |
| rs864745 | 7 | JAZF1 | T | C | 0.100 | Zeggini E et al, 2008(7) |
| rs896854 | 8 | TP53INP1 | T | C | 0.049 | Morris AP et al, 2012(35) |
| rs13266634 | 8 | SLC30A8 | t | c | -0.100 | Cho YS et al, 2011(36) |
| rs516946 | 8 | ANK1 | C | T | 0.086 | Morris AP et al, 2012(35) |
| rs17584499 | 9 | PTPRD | T | C | 0.450 | Tsai FJ et al, 2010(30) |
| rs2796441 | 9 | TLE1 | G | A | 0.068 | Morris AP et al, 2012(35) |
| rs7041847 | 9 | GLIS3 | a | g | 0.087 | Cho YS et al, 2011(36) |
| rs5015480 | 10 | HHEX | C | T | 0.140 | Morris AP et al, 2012(35) |
| rs10906115 | 10 | CDC123, CAMK1D | a | g | 0.082 | Cho YS et al, 2011(36) |
| rs10886471 | 10 | GRK5 | C | T | 0.110 | Li H et al, 2013(29) |
| rs5215 | 11 | KCNJ11 | C | T | 0.068 | Morris AP et al, 2012(35) |
| rs10830963 | 11 | MTNR1B | G | C | 0.095 | Morris AP et al, 2012(35) |
| rs1552224 | 11 | CENTD2 | A | C | 0.104 | Morris AP et al, 2012(35) |
| rs7961581 | 12 | TSPAN8, LGR5 | C | T | 0.090 | Zeggini E et al, 2008(7) |
| rs10842994 | 12 | KLHDC5 | C | T | 0.095 | Morris AP et al, 2012(35) |
| rs11634397 | 15 | ZFAND6 | G | A | 0.060 | Voight BF et al, 2010(40) |
| rs2028299 | 15 | AP3S2 | C | A | 0.100 | Kooner JS et al, 2011(31) |
| rs7403531 | 15 | RASGRP1 | T | C | 0.100 | Li H et al, 2013(29) |
| rs7178572 | 15 | HMG20A | G | A | 0.090 | Kooner JS et al, 2011(31) |
| rs7172432 | 15 | C2CD4A, C2CD4B | a | g | 0.101 | Cho YS et al, 2011(36) |
| rs8050136 | 16 | FTO | A | C | 0.160 | Zeggini E et al, 2007(38) |
| rs7202877 | 16 | BCAR1 | T | G | 0.113 | Morris AP et al, 2012(35) |
| rs4430796 | 17 | HNF1B | G | A | 0.104 | Cho YS et al, 2012(26) |
| rs12970134 | 18 | MC4R | A | G | 0.080 | Mahajan A et al, 2014(32) |
| rs12454712 | 18 | BCL2 | T | C | 0.040 | Z Samaan et al, 2015(34) |
| rs8090011 | 18 | LAMA1 | G | C | 0.030 | Matsuba R et al, 2015(39) |
| rs3794991 | 19 | GATAD2A | T | C | 0.110 | Prasad RB et al, 2015(27) |
| rs10401969 | 19 | CILP2 | C | T | 0.020 | Matsuba R et al, 2015(39) |
| rs11257655 | 10 | CDC123 | T | C | 0.060 | Mahajan A et al, 2014(32) |
| rs1558902 | 16 | FTO | a | t | 0.099 | Cho YS et al, 2011(36) |
| rs4858889 | 3 | SCAP | A | G | -0.170 | Saxena, R et al, 2013(17) |
